# Future avenues in *Drosophila* mushroom body research

**DOI:** 10.1101/lm.053863.123

**Published:** 2024-05

**Authors:** Ivy Chi Wai Chan, Nannan Chen, John Hernandez, Hagar Meltzer, Annie Park, Aaron Stahl

**Affiliations:** 1 Dynamics of Neuronal Circuits Group, German Center for Neurodegenerative Diseases (DZNE), Bonn, Germany; 2 Department of Developmental Biology, RWTH Aachen University, Aachen, Germany; 3 School of Life Science and Technology, Key Laboratory of Developmental Genes and Human Disease, Southeast University, Nanjing 210096, China; 4 Neuroscience Department, Brown University, Providence, Rhode Island 02906, USA; 5 Department of Molecular Cell Biology, Weizmann Institute of Science, Rehovot 7610001, Israel; 6 Department of Molecular Neuroscience, Weizmann Institute of Science, Rehovot 7610001, Israel; 7 Department of Physiology, Anatomy and Genetics, Centre for Neural Circuits and Behaviour, University of Oxford, Oxford, United Kingdom; 8 Neuroscience and Pharmacology, University of Iowa, Iowa City, Iowa 52242, USA

## Abstract

How does the brain translate sensory information into complex behaviors? With relatively small neuronal numbers, readable behavioral outputs, and an unparalleled genetic toolkit, the *Drosophila* mushroom body (MB) offers an excellent model to address this question in the context of associative learning and memory. Recent technological breakthroughs, such as the freshly completed full-brain connectome, multiomics approaches, CRISPR-mediated gene editing, and machine learning techniques, led to major advancements in our understanding of the MB circuit at the molecular, structural, physiological, and functional levels. Despite significant progress in individual MB areas, the field still faces the fundamental challenge of resolving how these different levels combine and interact to ultimately control the behavior of an individual fly. In this review, we discuss various aspects of MB research, with a focus on the current knowledge gaps, and an outlook on the future methodological developments required to reach an overall view of the neurobiological basis of learning and memory.

The anatomical structure known as the mushroom body (MB) was first discovered in bees and ants in the mid-nineteenth century and was initially considered to correlate in size with insect intelligence (Dujardin 1850; Forel 1874; Joescu 1909; von Alten 1910). More than a century later, the MB was identified as the center responsible for encoding associative memories (Menzel et al. 1974). The evolution of the MB field has taken us from mutating flies in order to identify structural phenotypes and learning impairments (Quinn et al. 1974; Heisenberg 1980) to the current state in which cutting-edge tools allow us to examine the molecular, physiological, and behavioral features of the brain at resolutions that were once inconceivable.

The remarkable progress made in MB research over the past decades lay the groundwork for the conceptual and technological developments that will support the next generation of MB researchers. Here, we reflect on some of the unresolved questions that we believe will accelerate the field in the upcoming years. We take this futuristic journey by gradually expanding our view on the MB circuit, starting with its development and synapse formation, through its intricate anatomical and functional connectivity, and culminating in its behavioral output (as summarized in Fig. 1). Finally, we consider the potential translational applications of this fascinating brain structure.

**Figure 1.**
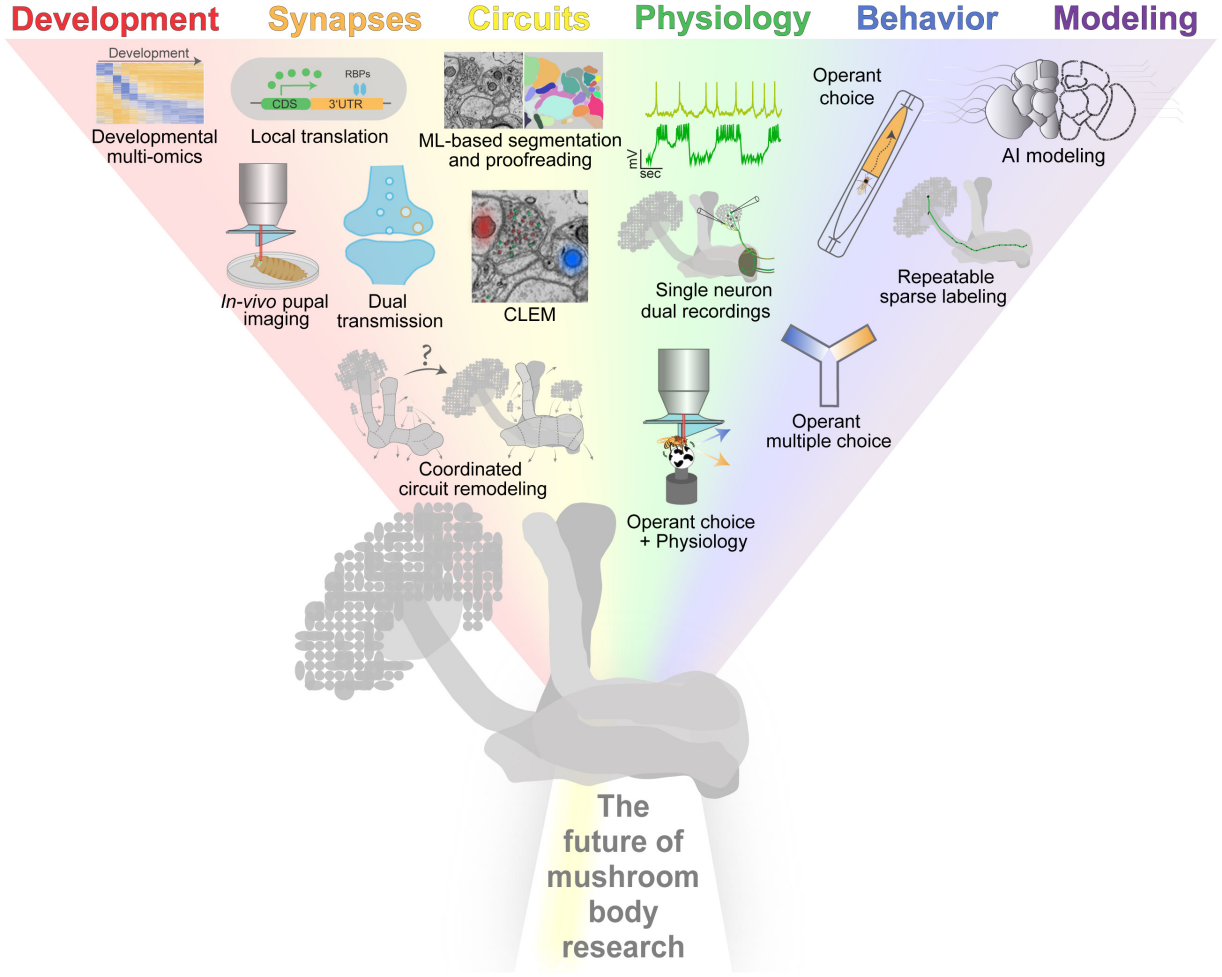
Forward-thinking outlook on learning and memory research in the mushroom body (MB). Uncovering how the MB circuit undergoes coordinated remodeling during metamorphosis, combined with developmental multiomics and in vivo pupal imaging, will advance our knowledge of neurodevelopmental principles. Studying dual transmission and activity-induced local translation will shed light on the neurotransmission mechanisms underlying learning and memory. Correlative light and electron microscopy (CLEM), alongside more accurate machine learning (ML)-based segmentation and proofreading, will promote our understanding of the MB circuit logic (EM image from the Kasthuri et al. 2015 data set). Single-neuron dual recordings will elucidate how the physiology of identified pre- and postsynaptic components change together. To delineate the circuit basis of motivated response, it is imperative to use assays designed to understand operant choice, how multiple rewards alter operant choice, and how operant choice relates to neural activity. Finally, refining artificial intelligence (AI) modeling and repeatable sparse labeling will enhance repeatability between experiments.

This review serves as a visionary overview, focused on our main fields of interest as young scientists in the fly community. Although *Drosophila* has been instrumental in driving significant breakthroughs in MB research, it is important to acknowledge that the structure and function of the MB varies across arthropods (Howse 1975; Strausfeld et al. 1998), with notable discoveries emerging from studies in non-*Drosophila* insects. For detailed coverage of the major milestones in MB research over the past decades, we direct readers to recent reviews (e.g., Aso and Rubin 2020; Boto et al. 2020; Modi et al. 2020; Puñal et al. 2021; Menzel 2022; Davis 2023; Lin 2023), as well as the various contributions within this special issue.

## How is the complex MB circuit assembled during fly development?

The function of the MB in learning and memory relies on the precise formation of its intricate neuronal circuit. Thus, delineating the molecular and cellular basis of MB development is crucial for obtaining a comprehensive understanding of its behavioral roles. Although tremendous progress has been made, we are far from reaching a holistic view of the events orchestrating MB development.

Initial MB development, occurring during the embryonic and larval stages, is relatively well-characterized in terms of the cell fate determination of its intrinsic neurons, the three types of Kenyon cells (γ-, α′/β′-, and α/β-KCs). However, very little is known about KC axon guidance, pathfinding, and branching into the vertical and medial lobes (Lin 2023). Following its initial establishment, the larval MB then undergoes massive rearrangements to accommodate the needs of an adult fly (Truman 1990). The most characterized remodeling process is the stereotypic pruning and regrowth of γ-KC axons (Lee et al. 1999; Yaniv and Schuldiner 2016). However, extrinsic members of the MB circuit, including the MB output neurons (MBONs) and the modulatory dopaminergic neurons (DANs), also display dramatic changes between larva and adult in terms of identity, number, and morphology. For example, the larval medial lobe is only innervated by four DANs (known as pPAMs), whereas the adult medial lobes are innervated by more than a hundred PAM-DANs. The stereotypical innervations by DANs and MBONs divide the KC axons to discrete compartments—16 in the adult MB but only 10 in the larva. Whereas for many years how this larva-to-adult transition occurs was largely a mystery, a recent, much-anticipated study provided comprehensive mapping of the MB circuit throughout metamorphosis (Truman et al. 2023). It demonstrated that MB metamorphosis combines cell death, recruitment of new neurons, and remodeling of neuronal processes within the MB or to non-MB targets (*trans*-differentiation). Along with the recently completed electron microscopy (EM)-based full-brain connectomes of both larvae and adult flies (Zheng et al. 2018; Scheffer et al. 2020; Dorkenwald et al. 2023; Lin et al. 2023; Schlegel et al. 2023; Winding et al. 2023), these resources are transforming the field, allowing new directions of experimental exploration. Thus, developmental research is expanding, from focusing on cell-intrinsic mechanisms to exploring how cell–cell interactions within the MB instruct coordinated circuit remodeling, as was already demonstrated for γ-KCs and the anterior paired lateral (APL) neuron (Mayseless et al. 2018; Meltzer and Schuldiner 2020). Furthermore, MB lobe assembly requires the spatial convergence of KCs, MBONs, and DANs within the different axonal compartments, but the temporal order by which they reach their target compartments, and whether they provide guidance for one another, is poorly understood (Bornstein et al. 2021; Lin et al. 2022; Lin 2023). The molecular events underlying the development of the calyx, in which KC dendrites wire with projection neurons (PNs) in a sparse, combinatorial manner, are also just beginning to be unraveled (Puñal et al. 2021). These findings in culmination could illustrate how MB circuit development shapes its function and, in turn, fly behavior.

Peering into the future, we anticipate technological innovations will facilitate the resolution of many current knowledge gaps. Importantly, the major advantage of *Drosophila* is its amazing toolbox, including countless driver lines that provide spatiotemporally regulated genetic handles to almost every cell type. A current limitation, however, is the dearth of specific drivers that are consistently expressed throughout pupal life, thus decreasing our ability to label or manipulate developing MB cells. Nonetheless, with constantly expanding driver collections and unceasing refinement of binary expression systems, we believe future efforts will solve this challenge. Current methods to sparsely label neuronal populations in a robust, predictable, and repeatable manner are also suboptimal (Lee and Luo 1999; Nern et al. 2015; Isaacman-Beck et al. 2020), and their improvement would push forward MB research, not just in the developmental context but in the field in general. Additional invaluable tools for developmental studies are CRISPR-based editing strategies, such as endogenous protein tagging, which allows tracking of protein localization, dynamics, and interactions throughout MB development at up to single-cell resolution (Sanfilippo et al. 2024). Rapidly evolving single-cell multiomics approaches, conducted along different developmental stages and potentially spatially resolved (Wang et al. 2022), will provide valuable resources for experimental studies, as well as the generation of novel reagents (Chen et al. 2023). EM reconstructions of pupal stages will uncover the dynamics of the MB connectome. Advanced neuroimaging techniques will propel our comprehension even further. Currently, imaging the developing MB within the intact pupa is impeded by the surrounding opaque fat bodies, thus limiting live imaging to ex vivo preparations (Rabinovich et al. 2015). However, with continuous improvements in super-resolution technologies and digital processing, in vivo pupal imaging is within reach.

Importantly, deciphering MB development will deepen our understanding of fundamental principles underlying the assembly of circuits of similar structural principles in higher organisms, such as the mammalian cerebellum (Farris 2011; Li et al. 2020). Furthermore, uncovering the principles underlying MB remodeling may provide certain mechanistic insights into neurodevelopmental conditions in humans, such as autism spectrum disorder and schizophrenia, which have long been associated with defects in neuronal remodeling (Cocchi et al. 2016; Sekar et al. 2016; Thomas et al. 2016).

## How do insights from MB synapses enhance our understanding of local protein synthesis?

Well-developed, mature neural systems regulate cognition functions through proper synaptic transmission. Synapses are the special structures that transmit information from one neuron to other neurons or effective cells. Neurotransmitters or neuropeptides are the mediators that transmit signals between cells. How these mediators are packaged into vesicles and then released into the synaptic cleft is not well-understood. KCs were reported to release both acetylcholine and neuropeptides (Lyutova et al. 2019), which are separately packaged into synaptic vesicles and dense-core vesicles. Using the MB as a model, we can investigate how different patterns of stimulation induce different release patterns.

Functional synaptic transmission requires the proper assembly of synaptic components. How the different synaptic proteins construct the complex synaptic structure is a crucial unresolved question. One fascinating element to consider is the translational aspect, as it is known that some synaptic proteins are translated at somatic regions and later transported to the synaptic regions, whereas others are locally translated (Hafner et al. 2019). How local translation of synaptic proteins is instructed (e.g., via activity-triggered site-specific translation) and whether local translation recruits RNA-binding proteins (RBPs) or relieves translational suppression from microRNAs (miRNAs) are all important questions that remain unanswered. The unique structure of the MB makes it an ideal model to study these questions, as KCs have long processes that are located far away from the soma.

A big obstacle for studying local synaptic translation is visualizing synaptic mRNAs, because their absolute number is much lower than that of somatic mRNAs. With recent developments in hybridization chain reaction (HCR) (Choi et al. 2018), it is now possible to detect synaptic mRNAs within MB regions. To investigate activity-induced transcription of synaptic genes in the MB, we can sequence and compare changes in mRNA levels in response to neuronal stimulation. Furthermore, to monitor activity-induced local protein synthesis at synaptic regions, a chemical tag can be recruited and knocked into the endogenous genes, and consequently nascent protein translation can be distinguished by identifying newly formed fluorescent signals (Wang et al. 2015), showing the activity-induced local translation of synaptic proteins. To identify potential RBPs and miRNAs that bind and regulate genes of interest, one can use in silico prediction tools (Chen et al. 2022). The expression pattern of RBPs can be studied by endogenously inserted fluorescent proteins. By using guide RNA-based knockouts (Port and Bullock 2016) or RNAi-based knockdown of RBPs, as well as miRNA sponge tools to block RBP binding to mRNAs (Fulga et al. 2015), we can examine how they regulate local translation of the synaptic proteins. Thus, these methods will advance us toward understanding the local protein regulation of KC synapses upon neural activation or synaptic plasticity.

## What is the wiring logic of the MB circuit underlying learning and memory?

The intricate orchestration of synaptic functions and plasticity within the MB is crucial for the formation and retention of long-term memories. But how different sensory information is integrated to initiate the much-needed synaptic plasticity at the KC–MBON synapses remains a fundamental question. In the MB main calyx, more than 2000 KCs receive combinatorial inputs from 52 types of olfactory PNs, representing odor information in a sparse and unique code modulated by the inhibitory action of the APL (Honegger et al. 2011; Gruntman and Turner 2013; Lin et al. 2014; Prisco et al. 2021). Although it was previously believed that random PN-to-KC connections optimize odor discrimination performance (Litwin-Kumar et al. 2017), multiple lines of evidence have suggested the presence of potential structure in the connections. Density maps of neuropil distribution generated from light microscopy (LM) preparations have revealed a conserved spatial distribution pattern of PN and KC types in the calyx across individuals (Wong et al. 2002; Jefferis et al. 2007; Lin et al. 2007). Further advancement of serial section transmission EM (ssTEM) led to the first full MB connectome of a first-instar *Drosophila* larva with synaptic resolution (Eichler et al. 2017). Although most KCs integrate random combinations of inputs, Eichler et al. found that a subset receives stereotyped inputs from single PNs in the larval MB. In 2018, Zheng et al. produced a complete EM volume of a female adult fly brain (FAFB) at synaptic resolution with the use of ssTEM. By sparsely and manually tracing the FAFB EM data set, Zheng et al. (2022) described structures in the wiring logic between food-odor-responding PN types and α′/β′- and α/β-KCs. As a multisensory integration center, the MB calyx also integrates inputs from visual, gustatory, and thermosensory regions, facilitating multimodal-associative learning (Guo and Guo 2005; Vogt et al. 2014; Kirkhart and Scott 2015; Yagi et al. 2016; Li et al. 2020).

Different sensory information is associated within the MB lobes through complex neural mechanisms. The current memory acquisition model suggests that, upon associative learning, the sensory inputs from KC axons coincidentally meet with the appetitive or aversive reinforcing DAN synapses, which will in turn modify the synaptic strength between KCs and MBONs, leading to sensory input-specific attraction or avoidance behavior. By using advanced genetic approaches such as the split-Gal4 system, the laminar arborization of the KC axons and the compartmental innervation patterns of DANs and MBONs were discovered (Tanaka et al. 2008; Mao and Davis 2009; Aso et al. 2014). Functionally, the individual compartments could exhibit different rates of memory acquisition and decay, as well as encode different memory types (Cohn et al. 2015; Aso and Rubin 2016; Perisse et al. 2016). By fully reconstructing a male MB α lobe with ssTEM, Takemura et al. (2017) revealed the surprisingly direct DAN–MBON connections and the complex synaptic structures between KCs and DANs. Functionally activating DANs alone can weaken memory recall by depolarizing the postsynaptic MBON. In 2020, Scheffer et al. presented the Hemibrain, a dense reconstruction of the central brain of an adult female fly with the use of focused ion beam scanning EM (FIB-SEM). With the higher spatial resolution offered by FIB-SEM, analysis of the Hemibrain revealed new components of the MB circuit, including vast visual input from the local visual interneurons to two KC classes, direct feedback circuit from MBONs to DANs, and “atypical” MBONs with direct connections to descending neurons (Li et al. 2020). Last year, the much-anticipated whole-brain connectome was produced from the initial FAFB EM data with the help of ML techniques (now known as FlyWire) (Dorkenwald et al. 2023; Lin et al. 2023; Schlegel et al. 2023). Along with the recent whole-brain connectome of the first-instar larva (Winding et al. 2023), these resources are opening new doors to identify the downstream partners of individual MBONs.

Connectomes have provided us with an unprecedented forward leap to deepen our understanding of the MB circuit. However, the current adult MB connectomes are of two female Canton-S flies of 5–7 d posteclosion, thus do not encompass the differences in the MB circuit due to sexual dimorphism, aging, and different genotypes. To enable comparison of connectomes between more individual flies across sex and ages, even faster and more accurate ML-based segmentation and proofreading techniques are necessary. This will also help address a wider variety of questions such as how learning or aging modifies the MB circuit. On top of that, it will allow us to investigate how evolution shapes the MB by comparing the connectomes of different *Drosophila* species and other arthropods (Ellis et al. 2023).

However, a connectome is only a snapshot of a highly dynamic system. Without investigating neural plasticity, network dynamics, and neuromodulation, one cannot get closer to explaining how a fly will behave at a given time point. One way to address these factors is to combine the analysis of cellular and biophysical properties of the neurons with information about their connections. For instance, correlative fluorescence immunostaining with EM can be used to identify the expression of specific proteins in individual neurons as well as their connectivity. Furthermore, a map of the subcellular distribution and properties of key molecular players, such as voltage-gated ion channels, in different MB neurons will improve our understanding of possible network dynamics (Gratz et al. 2019; Ravenscroft et al. 2020). Moreover, the current connectomes focus solely on connectivity that arises from chemical synapses of neurons, yet gap junctions between neurons are also known to play a key role in noise reduction and neural synchronization in the MB (Liu et al. 2016; Gutierrez and Wang 2023). Finally, although the interplay between neurons and glia also shapes the MB response and behavioral output, glia in the MB are yet to be fully reconstructed in the EM data sets (Park et al. 2022).

## How are the input signals integrated within the MB?

Although the connectome provides us with predictive connectivity, it is necessary to functionally validate these connections using voltage/calcium imaging or electrophysiology. Ideally, this is done by recording from at least two neurons at once—perhaps by combining sparse labeling to drive expression of genetically encoded voltage indicators in a few cells that are sufficiently spatially separated to resolve single unit activity or dual whole-cell recordings (Gaudry et al. 2013; Isaacman-Beck et al. 2020). One key advantage of whole-cell patch clamp electrophysiology is the ability to inject precise amounts of current and observe how the cell(s) respond. Using this technique, one could inject physiologically relevant patterns of current into one cell and read how signals are integrated out of the other.

Leveraging these techniques will allow us to resolve outstanding questions about the network-level computations performed by the MB. For instance, we know that subsets of DANs that project to a single compartment are heterogeneous, but do individual neurons exert distinct signals or are they functionally redundant? Do all DANs that project to a single compartment convey a population code derived from ensemble activity? Or do these individual DANs operate in a parallel noninteracting fashion? The organizational structure of the MB also provides us with the ideal system to understand how a variety of input signals are integrated by a sparse number of output neurons or MBONs. Each compartment converges its inputs onto a countable number or sometimes a single MBON, which can directly influence behavioral output. This convergent architecture allows us to resolve questions such as: How are these signals integrated by the MBONs? Are these computations nonlinear? Understanding how these operations are performed on a physiological level by investigating the functional connectivity between neurons will allow us to decode the network-level computations performed in the MB. There is a strong possibility that these motifs of physiological operations performed by the MB are conserved across species. Furthermore, because it is currently impossible to perform in vivo whole-cell patch clamp of midbrain dopamine neurons in awake behaving animals, the value of investigating these questions in *Drosophila* becomes apparent.

A further benefit of recording from neurons projecting into the MB is that it will allow us to determine physiologically relevant patterns of activity that will refine our understanding of the computational operations performed in the MB. These patterns of activity can also be leveraged for directed experimental design. For instance, there are many examples in which specific patterns of stimulation can lead to differential outcomes of behavior (McGinnis et al. 2016), but why and how different types of stimulation produce distinct behaviors has largely been unexplored. Perhaps different frequencies of optogenetic stimulation activate specific subsets of downstream neurons that exhibit resonance or are tuned to a precise spike frequency.

As the field further scrutinizes the anatomical and molecular features of single neurons, it is important to assess whether the proper tools are available to address the questions at the level of detail we are approaching. Subcellular localization of proteins is critical for proper neuronal physiology and function, as the spatial organization of these proteins contributes to different neuronal encoding properties (Sheng et al. 1992; Steward 1997). Previous studies have also demonstrated spatially compartmentalized activation within individual neurons, such as dendritic branch– and spine-specific calcium influx (Yuste and Denk 1995; Cichon and Gan 2015; Bilz et al. 2020). Although we can appreciate these subtleties, there are few tools available for compartment-specific targeting of protein expression. For instance, by tethering *GtACR* (the commonly used protein for light-induced inhibition of neurons) (Emiliani et al. 2022) to a receptor (e.g., GABA_A_R or AkhR), we could silence specific types of inputs instead of broadly silencing the entire neuron. This strategy could allow for compartment- or even synapse-specific silencing of the neuron, allowing unaffected compartments to propagate signals normally.

## How does individuality in motivated response reflect in learning and memory?

The MB is commonly used as a model to understand behavioral choice focusing on aversive learning and memory (Akalal et al. 2006; Berry et al. 2008; Busto et al. 2010; Saitoe et al. 2022), aimed to delineate how animals learn via classical conditioning to avoid odors or cues (conditioned stimulus, CS) paired with shock or other aversive stimuli (unconditioned stimulus, US). Nonassociative paradigms offer the ability to study innate behavioral responses in flies (Kacsoh et al. 2015) and neural circuit function in bees (MaBouDi et al. 2017). *Drosophila* can also be trained to associate odors with the experience of consuming sucrose or ethanol intoxication (Tempel et al. 1983; Krashes and Waddell 2008; Kaun et al. 2011), with subsequent testing whether flies remember to seek paired odors in the absence of sucrose or ethanol. Classical conditioning approaches allow us to study how learning acquisition transitions into memory consolidation, and whether those memories are forgotten or stored as for the long term. However, the conditions of classical conditioning in the laboratory often do not examine more complex behavioral decision-making for US that is closer to what animals exhibit in nature. To date, very little is known about the behavior of *Drosophila* in natural contexts.

The US that guides classical conditioning includes stimuli such as water, food, sugar, and, for some individuals, drugs, which comprise broadly agreed-upon rewarding stimuli for animals. In *Drosophila*, there is evidence for the circuit basis of sucrose and food memory, relief from aversive experience, drug memory, and even successful copulation (Hernandez et al. 2023). Few studies have characterized the trajectory of how individual flies learn, via instrumental conditioning, to self-administer rewards. Tractable behavioral assays (Ja et al. 2007; Lau et al. 2017; Wiggin et al. 2021; Croteau-Chonka et al. 2022) to study instrumental learning allow researchers to identify and characterize how animals express motivation for the US. In this context, motivation could be characterized as an animal's willingness to perform or avoid behaviors that result in US exposure. Understanding how these behaviors are shaped through high-content behavioral analysis will inform how the memory is developed. To understand more complex behaviors, it is important that researchers develop assays that allow flies to perform natural behaviors (e.g., entering regions of an apparatus that are perceivably different from other regions, or engaging with substrates or objects that flies find in nature), which result in gaining immediate access to US. The goal in designing these assays should be to investigate how instrumental conditioning uncovers the individuality in learning, how the internal state alters learning via instrumental conditioning, and, ultimately, which circuits contribute to individuality in the expression of motivation.

Although the *Drosophila* model system offers accessible neurogenetic tools in identifiable circuits, one pitfall is the inability to monitor neural activity while animals are performing more natural instrumental behaviors. Developing better tools to record neural activity while animals learn via instrumental or classical conditioning could provide unique insights into how individual neurons function distinctly in these contexts. These tool advancements could also promote our understanding of *Drosophila* larval (Saumweber et al. 2018; Wong et al. 2021; Thoener et al. 2022) and bee learning (Pahlke et al. 2021; Lafon et al. 2022; MaBouDi et al. 2023). Delineating how individual identifiable neurons function in different learning contexts could allow us to develop targeted treatments when animals exhibit maladaptive behavioral motivation (e.g., consuming drugs to the point of intoxication).

## What is the next step in the evolution of disease modeling using the MB?

*Drosophila* in general, and the MB specifically, remains a powerful genetic model to replicate the molecular, morphological, and, to a limited extent, behavioral characteristics associated with neuropathological disorders. Such characteristics were identified through classical assays, thus becoming the foundation of disease modeling of both neurodevelopmental disorders such as schizophrenia, neurofibromatosis type 1, and Fragile X syndrome, as well as neurodegenerative diseases such as Alzheimer's and Parkinson's (Iijima et al. 2004; McBride et al. 2005; Lu and Vogel 2009; Buchanan and Davis 2010; Narayanan and Rothenfluh 2016; Machado Almeida et al. 2021). Currently, a new generation of techniques is leading to an innovative foundation utilizing machine learning. A key variable associated with any screen is the speed of throughput. Machine learning efforts facilitate the identification of subtle morphological changes, as well as significantly increase the speed of processing of large data samples (Diez-Hermano et al. 2020). Future efforts will combine these assays with genetic screens to validate human and fly orthologs of disease-causing genes (Singhal and Jaiswal 2018; Deal and Yamamoto 2019).

The current RNAi catalogs contain a vast amount of targeted knockdown approaches for the investigation of nearly all *Drosophila* genes (Dietzl et al. 2007; Zirin et al. 2020). Although this is a powerful tool for basic research, translational research requires the next step in an individualistic approach. So far, more than 1000 genes underlying intellectual disabilities alone were identified (Zirin et al. 2020), and although many patient-derived mutations have been previously generated, the library of mutations implicated in neurological diseases still needs to be expanded to model new pathophysiologies. Patient-derived sequencing will generate new targeted and streamlined databases for disease investigation. The CRISPR–Cas9 system is capable of generating large-scale screening tools targeted to specific genetic manipulations (Meltzer et al. 2019; Port et al. 2020; Koreman et al. 2021). Forward and reverse genetic screens will elucidate previously unidentified phenotypes that cause disease states within *Drosophila*.

Although *Drosophila* contains homologs to ∼75% of human disease–causing genes, many differences have plagued therapeutic approaches for screening within *Drosophila* (Reiter et al. 2001; Bier 2005). At the most basic levels, including molecular pathways and neuronal circuit functionality, *Drosophila* has served as a proxy to identify novel disease-causing molecular mechanisms and their consequential circuit aberrations (Shulman et al. 2003; Lenz et al. 2013; McGurk et al. 2015). However, key differences in anatomical and metabolic properties have made *Drosophila* a difficult tool to model therapeutics (Rand 2010). The most obvious limitations arise in tissue type differences, such as the absence of the substantia nigra within *Drosophila* to study Parkinson's disease, although DANs innervating the MB have served as a key model to identify pathophysiological characteristics of the disease (e.g., White et al. 2010; Riemensperger et al. 2013). A major obstacle of therapeutic research has been the differences between humans and flies in both pharmacokinetics and pharmacodynamics. Although these shortcomings may elicit negative results, the efficacy of *Drosophila* as an intermediary screening tool for therapeutic targets between mammalian cell cultures and rodents remains a viable tool (Pandey and Nichols 2011).

Genetic screens can be combined with machine learning to analyze and quantify the morphological, circuit, and behavior-level changes associated with diseases modeled in *Drosophila.* Deep neural network architectures based on the full-brain connectome will help researchers identify key circuits, many of which have afferent or efferent connections to the MB. This morphometric approach will analyze branching patterns, the number of connections, and their shape to compare morphologies in health versus disease states. Notably, with only 20% of the current *Drosophila* connectome identified within the current literature, it will be important to elucidate the function of the remaining 80% (Scheffer et al. 2020). Using this new information will deepen our understanding of classically studied diseases, but also potentially give rise to new avenues of research. Elucidating the incredible complexity of the MB circuitry and identifying the heterogeneity of connections has shown the potential for further modeling addiction (Jovanoski et al. 2023), mild forms of head trauma (Behnke et al. 2021), and chronic stress-induced learning deficits (Jia et al. 2021). Advanced activity monitoring assays (e.g., Kain et al. 2013; Haberkern et al. 2022), combined with connectomics data, will allow researchers to gain new insights into the underpinnings of neurological disorders for new translational approaches to be tested.

## Concluding remarks

In the 25 years that have passed since the previous special issue focusing on the MB was published in *Learning & Memory* (https://learnmem.cshlp.org/content/5/1.toc), our understanding of MB anatomy, connectivity, and function has considerably evolved. This was largely due to the development of the tools and techniques mentioned throughout this review, which have allowed *Drosophila* researchers to gain access to the molecular, anatomical, and physiological features of single neurons at unparalleled levels of resolution. However, although we can appreciate that neurons are more complex and heterogeneous than ever before, has our understanding of the computational operations they perform within the MB evolved at a similar pace? In other words, we can now appreciate the intricacies of the building blocks of the brain at different functional levels, but has this enhanced our overall understanding of the network-level computations performed in the MB and how these directly relate to behavior?

In the past few decades, *Drosophila* MB researchers have taken a steadfast approach in identifying precise subsets of molecules and neurons involved in modulating behavior. By manipulating the relative abundance of specific proteins or activating/silencing neuronal activity, we have obtained a comprehensive list of molecules and neurons critical for MB-related behaviors—namely, associative learning. However, what we currently lack is a mechanistic understanding of how these molecules tune neuronal physiology and the physiological principles dictating the neurons themselves. Developing this framework will allow us to gain a deeper understanding of the computational operations performed in the brain, and how these ultimately modulate behavior. Although the fly could never capture the complexities of human cognition and behavior, its ability to perform complex behaviors, despite the reduced numerical complexity of its brain, provides *Drosophila* researchers the unique opportunity to develop such a framework and determine how it directly relates to MB-associated behaviors. Although the current genetic toolkit is vast, the ability to manipulate molecules and neurons with a greater level of nuance, such as subcellular targeting of proteins, will be critical for determining the precise mechanisms by which the MB operates. Understanding the neurobiological principles of simpler brains will provide deeper insight into how evolutionarily distant animals perform similar behaviors.
